# Myocardial Infarct Size by CMR in Clinical Cardioprotection Studies: Insights From Randomized Controlled Trials

**DOI:** 10.1016/j.jcmg.2017.01.008

**Published:** 2017-03

**Authors:** Heerajnarain Bulluck, Matthew Hammond-Haley, Shane Weinmann, Roberto Martinez-Macias, Derek J. Hausenloy

**Affiliations:** a The Hatter Cardiovascular Institute, Institute of Cardiovascular Science, University College London, United Kingdom; b The National Institute of Health Research University College London Hospitals Biomedical Research Center, London, United Kingdom; c National Heart Research Institute Singapore, National Heart Center Singapore, Singapore, Singapore; d Cardiovascular and Metabolic Disorders Program, Duke-National University of Singapore, Singapore, Singapore

**Keywords:** cardiovascular magnetic resonance, myocardial infarct size, primary percutaneous coronary intervention, randomized controlled trial, sample size, ST-segment elevation myocardial infarction, AAR, area at risk, CMR, cardiac magnetic resonance, FWHM, full width half maximum, GBCA, gadolinium-based contrast agent, Gd-DOTA, gadoterate meglumine, Gd-DTPA, gadopentetate dimeglumine, LGE, late gadolinium enhancement, LV, left ventricular, MI, myocardial infarct, MSI, myocardial salvage index, MVO, microvascular obstruction, PPCI, primary percutaneous coronary intervention, RCT, randomized controlled trial, STEMI, ST-segment elevation myocardial infarction

## Abstract

**Objectives:**

The aim of this study was to review randomized controlled trials (RCTs) using cardiac magnetic resonance (CMR) to assess myocardial infarct (MI) size in reperfused patients with ST-segment elevation myocardial infarction (STEMI).

**Background:**

There is limited guidance on the use of CMR in clinical cardioprotection RCTs in patients with STEMI treated by primary percutaneous coronary intervention.

**Methods:**

All RCTs in which CMR was used to quantify MI size in patients with STEMI treated with primary percutaneous coronary intervention were identified and reviewed.

**Results:**

Sixty-two RCTs (10,570 patients, January 2006 to November 2016) were included. One-third did not report CMR vendor or scanner strength, the contrast agent and dose used, and the MI size quantification technique. Gadopentetate dimeglumine was most commonly used, followed by gadoterate meglumine and gadobutrol at 0.20 mmol/kg each, with late gadolinium enhancement acquired at 10 min; in most RCTs, MI size was quantified manually, followed by the 5 standard deviation threshold; dropout rates were 9% for acute CMR only and 16% for paired acute and follow-up scans. Weighted mean acute and chronic MI sizes (≤12 h, initial TIMI [Thrombolysis in Myocardial Infarction] flow grade 0 to 3) from the control arms were 21 ± 14% and 15 ± 11% of the left ventricle, respectively, and could be used for future sample-size calculations. Pre-selecting patients most likely to benefit from the cardioprotective therapy (≤6 h, initial TIMI flow grade 0 or 1) reduced sample size by one-third. Other suggested recommendations for standardizing CMR in future RCTs included gadobutrol at 0.15 mmol/kg with late gadolinium enhancement at 15 min, manual or 6-SD threshold for MI quantification, performing acute CMR at 3 to 5 days and follow-up CMR at 6 months, and adequate reporting of the acquisition and analysis of CMR.

**Conclusions:**

There is significant heterogeneity in RCT design using CMR in patients with STEMI. The authors provide recommendations for standardizing the assessment of MI size using CMR in future clinical cardioprotection RCTs.

## Graphical abstract

**Figure:**
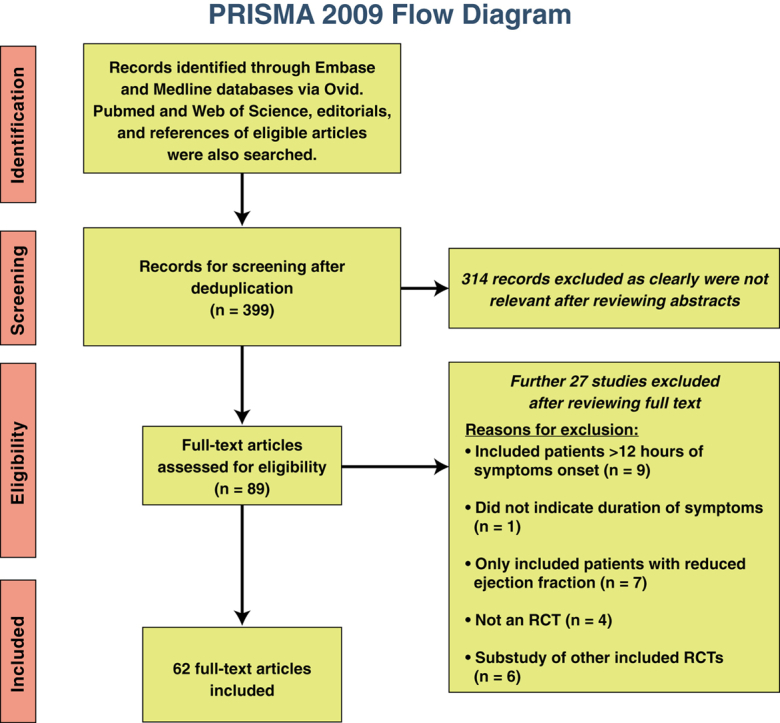

Since the introduction of primary percutaneous coronary intervention (PPCI) for the treatment of acute ST-segment elevation myocardial infarction (STEMI), mortality has improved substantially (1), but the morbidity associated with post–myocardial infarct (MI) heart failure remains significant (2). Cardiac magnetic resonance (CMR) is increasingly being used to quantify MI size in randomized controlled trials (RCTs) investigating novel cardioprotective therapies targeting myocardial reperfusion injury to reduce MI size in patients with STEMI treated with PPCI (3).

Late gadolinium enhancement (LGE) by CMR is considered the gold standard for MI size quantification (4). MI size (5), microvascular obstruction (MVO) (6), and myocardial salvage (7) assessed by CMR performed in the first few days post-PPCI have all been shown to be strongly prognostic. As a result, CMR is increasingly being used for surrogate endpoints in RCTs (3). A recent meta-analysis of 2,632 patients from 10 RCTs found that MI size measured by CMR or single-photon computed tomography within 1 month post-PPCI showed that for every 5% increase in MI size, there was a 20% increase in the relative hazard ratio for 1-year hospitalization for heart failure and all-cause mortality (5).

Despite CMR endpoints being quite robust and their ability to keep sample size small (3), there is limited guidance on its use. In this study, we reviewed all published RCTs in this field so far, and we provide recommendations for standardizing the use of CMR in future clinical cardioprotection RCTs.

## Methods

**Figure 1:**
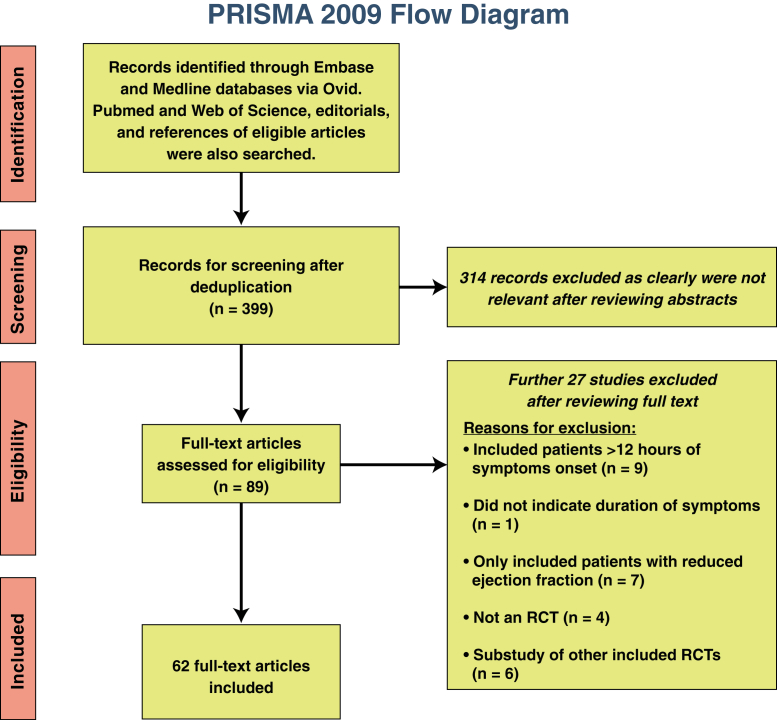
Preferred Reporting Items for Systematic Reviews and Meta-Analyses Flow Diagram This figure shows the process of identifying, screening, and selecting the randomized controlled trials (RCTs) included in this study. Of 399 studies screened, 62 RCTs eventually met the inclusion criteria. PRISMA = Preferred Reporting Items for Systematic Reviews and Meta-Analyses.

We performed a comprehensive systematic search in the MEDLINE and Embase databases via Ovid up to November 23, 2016. PubMed and the Web of Science, editorials, and the reference lists of included RCTs were also screened. Further details of the search terms and the study screening and selection as per the Preferred Reporting Items for Systematic Reviews and Meta-Analyses are provided in the Online Appendix. The flow diagram is shown in Figure 1.

The inclusion criteria were as follows: 1) RCTs investigating cardioprotective strategies aimed at reducing MI size; 2) including patients presenting within 12 h of symptom onset; 3) MI size measured by CMR; and 4) RCTs with full-text reports in English. The exclusion criteria were as follows: 1) RCTs selecting patients on the basis of admission ejection fraction; 2) RCTs performing post hoc analysis on other included RCTs; 3) observational studies; and 4) RCTs only reporting left ventricular (LV) ejection fraction by CMR.

### Study selection and data extraction

Four authors (H.B., M.H.-H., S.W., R.M.M.) independently identified suitable RCTs from the screened reports and extracted all data. Data on study characteristics, patient eligibility criteria, CMR scanner and contrast agent used, MI size quantification technique used, and mean MI size and SD in the control arm were collected. RCTs reporting both acute MI size and follow-up MI size (≥1 month) were recorded. The primary outcome of interest was MI size, expressed as a percentage of LV volume or mass (%LV).

### Statistical analysis

Categorical data are reported as frequencies and percentages. For sample size calculation, to ensure normally distributed MI size in the control arms, only RCTs reporting mean MI size as %LV were included, and an unpaired Student *t* test was used. Only RCTs including STEMI in all coronary territories were included, and they were grouped according to duration of symptoms (<6 or <12 h) and TIMI (Thrombolysis In Myocardial Infarction) flow grade 0 or 1 or 1 to 3 in the culprit arteries pre-PPCI if at least 3 RCTs were present in the subgroups. The mean and SD from each control arm were weighted against its respective sample size to obtain representative pooled mean acute and follow-up MI sizes using RevMan version 5.2 (Nordic Cochrane Center, Copenhagen, Denmark). Sample-size calculation was performed for 90% power, a 2-sided alpha value of 0.05, and expected effect sizes of 20%, 25%, and 30% using Stata/IC version 12.1 (StataCorp LP, College Station, Texas). The expected sample sizes were provided for each group after accounting for potential dropouts.

## Results

Our initial search identified 399 reports and 62 RCTs, involving 10,570 patients, that met the inclusion criteria. Details of the 62 included RCTs are provided in Online Table 1.

### Characteristics of the RCTs

**Figure 2:**
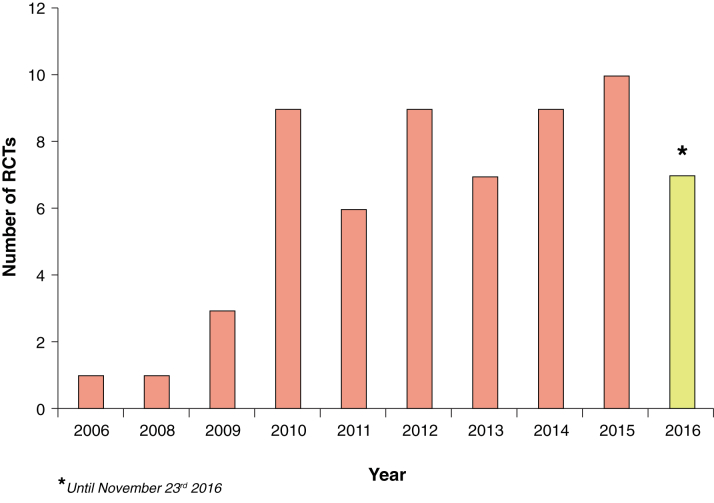
Number of Randomized Controlled Trials Published Each Year Since 2006 This bar chart shows the gradual increase in the number of randomized controlled trials (RCTs) published each year over the past 11 years.

The number of RCTs using CMR has steadily increased since 2009 (Figure 2). There was an average of 8 RCTs reported per year between 2010 and 2016. The majority of RCTs came from Europe (41 of 62 [66%], 6,880 patients), and 35 of 62 (56%) were single-center RCTs.

MI size as %LV was reported in 44 RCTs (71%). Five RCTs (8%) reported MI size as percentage of the area at risk (AAR), 5 (8%) in grams, and 7 RCTs (12%) reported myocardial salvage index (MSI).

**Figure 3:**
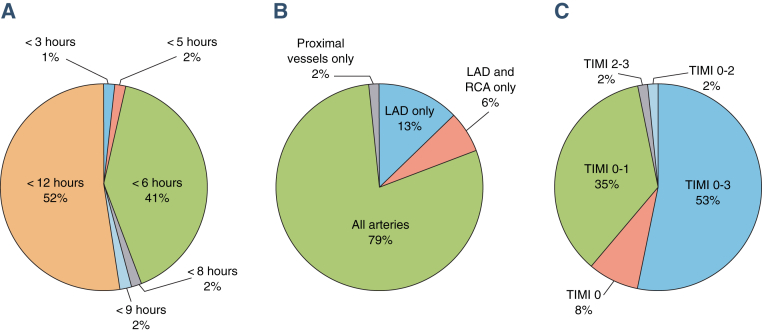
Distribution of RCTs by Inclusion of Patients on the Basis of Duration of Symptoms, Infarct-Related Artery Included, and TIMI Flow Grade Pre–Primary Percutaneous Coronary Intervention These 3 pie charts show the percentage of randomized controlled trials (RCTs) including **(A)** patients on the basis of duration of symptoms, **(B)** infarct-related artery, and **(C)** TIMI (Thrombolysis in Myocardial Infarction) flow grade pre–primary percutaneous coronary intervention (PPCI). The majority of the RCTs included patients presenting within 12 h of symptom onset, with ST-segment elevation myocardial infarction in all coronary territories, and with all pre-PPCI TIMI flow grades. LAD = left anterior descending coronary artery; RCA = right coronary artery.

The majority of RCTs included: 1) patients presenting within 12 h of symptom onset (32 of 62 [52%]); 2) STEMI in all epicardial territories (49 of 62 [79%]); and 3) patients with all TIMI flow pre-PPCI (33 of 62 [53%]), as shown in Figure 3.

Forty-six of the RCTs (74%) used 1.5-T scanners, and a minority (2 RCTs [3%]) used 3-T scanners. Four RCTs (7%) used a combination of 1.5- and 3-T scanners. Ten RCTs (16%) did not specify the field strength of the CMR scanners used, and 8 of 10 (80%) of those were multicenter RCTs.

Thirty-six of the RCTs (58%) used single CMR vendors, and 7 RCTs (11%) used multivendor scanners at different recruitment sites. Nineteen RCTs (31%) did not specify the vendors used, the majority of which (15 RCTs [75%]) were multicenter RCTs.

Paired acute and follow-up CMR was performed in 26 RCTs (42%), acute CMR only in 24 RCTs (39%), and follow-up CMR only in 12 RCTs (19%).

### Gadolinium-based contrast agent and dose

Gadopentetate dimeglumine (Gd-DTPA) (Magnevist, Bayer Healthcare, Berlin, Germany), gadoterate meglumine (Gd-DOTA) (Dotarem, Guerbet, Roissy CdG Cedex, France), and gadobutrol (Gadovist, Bayer Healthcare) were used in 18 (29%), 12 (19%), and 9 (14%) of the 62 RCTs, respectively. Nineteen of 62 RCTs (31%) did not specify the gadolinium-based contrast agent (GBCA) used, of which 15 of 19 (79%) were multicenter RCTs. Online Table 2 summarizes the doses of the GBCAs and the reported timing for LGE acquisition post-contrast. The most common doses for Gd-DTPA (Magnevist), gadobutrol (Gadovist), and Gd-DOTA (Dotarem) were similar at 0.20 mmol/kg, with LGE acquisition starting at 10 min onward.

### Timing of CMR acquisition

**Figure 4:**
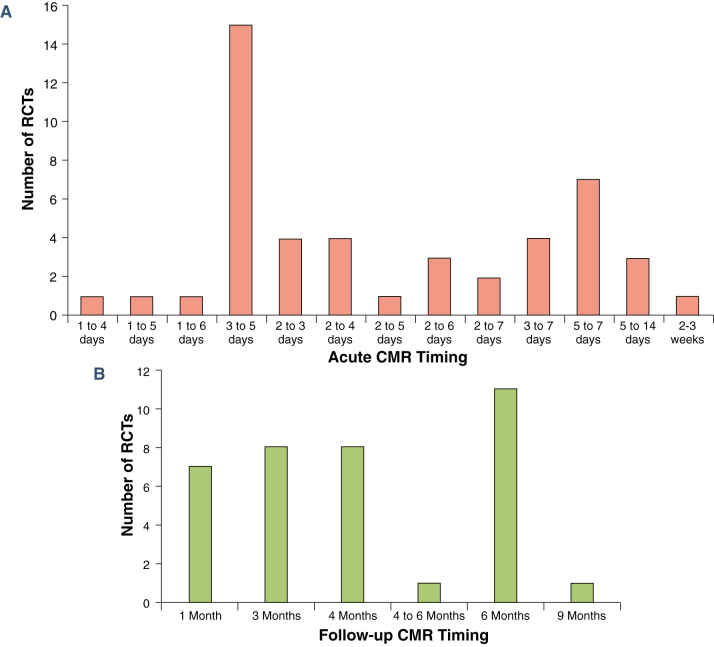
Timing of Acute and Follow-Up CMR These 2 bar charts show the distribution of the timings of the **(A)** acute and **(B)** follow-up cardiac magnetic resonance (CMR) in the randomized controlled trials (RCTs) included. There was a wide range of timings for both scans, and the most common timings were 3 to 5 days for the acute scan and 6 months for the follow-up scan.

The distribution of the timings to acquire acute and follow-up CMR is shown in Figure 4. Of 50 RCTs with acute CMR acquisition, only 1 did not specify the exact timing of the scan. There was a wide range of timings to acquire the acute CMR scan, with the majority (82%) acquired within the first 7 days post-PPCI and the most common timing being 3 to 5 days post-STEMI (15 of 50 RCTs [30%]). There was also a wide range of timings (1 to 9 months) to acquire follow-up MI size, with the most common timing being 6 months (11 of 37 RCTs [30%]).

### MI size analysis

Seventeen of 25 of the multicenter RCTs (68%) specified using a CMR core laboratory. The majority of the RCTs (29 of 62 [47%]) used specialist software (Qmass, Medis Medical Imaging Solutions, Leiden, the Netherlands; CVI42, Circle Cardiovascular Imaging Inc., Calgary, Canada; Segment, Medviso, Lund, Sweden; and CAAS, Pie Medical Imaging B.V., Maastricht, the Netherlands) for MI size quantification, 7 RCTs (11%) used the scanners’ own software, and 5 of 62 (8%) used shareware (ImageJ, National Institutes of Health, Bethesda, Maryland; OsiriX, Geneva, Switzerland). Twenty-one of 62 RCTs (34%) did not specify the analysis tool used, of which 9 of 21 (43%) were multicenter RCTs.

**Figure 5:**
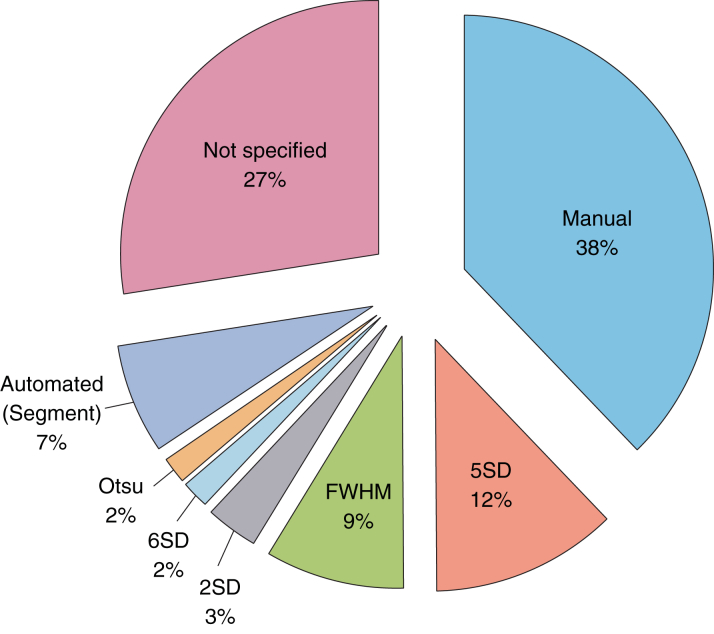
Quantification Techniques for MI Size The most common myocardial infarct (MI) size quantification method was manual delineation, followed by 5-SD and full width half maximum (FWHM). However, 27% of randomized controlled trials did not specify the method used.

Manual contouring was the most commonly used method (22 of 62 RCTs [36%]), followed by 5-SD (8 of 62 RCTs [13%]) and full width half maximum (FWHM) (6 of 62 RCTs [10%]). Sixteen of 62 RCTs (27%) did not specify the method used for MI quantification, of which 9 of 16 (56%) were multicenter RCTs. Figure 5 shows the distribution of the quantification techniques for MI size used in these RCTs.

### Distribution of culprit vessel and TIMI flow pre-PPCI

Among the RCTs including all culprit vessels (n = 6,020), 45% of the patients had STEMI in the left anterior descending coronary artery territory. Among the RCTs including all TIMI flow grades (n = 3,229), 73% of patients had TIMI flow grades of 0 or 1 pre-PPCI.

### Effect size for reduction in MI size in RCTs with positive results

Seventeen RCTs were positive for a reduction in MI size. Of the RCTs reporting MI size as %LV, the median reduction in acute MI size was 34% (mode 35%; range 14% to 38%). As for chronic MI size, both the median and mode reduction in MI size were 18% (range 14% to 46%).

### MI size in the control ARM

RCTs reporting mean ± SD, including STEMI presenting within 6 or 12 h, in all coronary territories, and with TIMI flow grades of 0 or 1 or 0 to 3 pre-PPCI were selected to obtain representative weighted mean MI size, expressed as %LV in the control arm for both acute (20 RCTs) and follow-up (18 RCTs) CMR (summarized in Online Tables 3 and 4). The expected sample sizes on the basis of 20%, 25%, and 30% reductions in MI size are summarized in Table 1. The expected sample sizes are provided on the basis of 90% power and a 2-sided alpha of 0.05.

The estimated sample size was smallest for RCTs including patients with STEMI within 6 h of symptom onset and with pre-PPCI TIMI flow grades of 0 or 1 and was largest for RCTs recruiting patients within 12 h of symptom onset and with any TIMI flow grades pre-PPCI, and this was driven by a wider SD in the latter group. For the same effect size, RCTs planning to use chronic MI would require on average 30% more patients than if acute MI were chosen as the endpoint.

### Dropout rates

In RCTs using CMR for the primary endpoint, the average dropout rate was 9% for RCTs using acute CMR only, 13% for RCTs acquiring follow-up CMR only, and 16% for those with paired acute and follow-up scans.

## Discussion

On the basis of the 62 RCTs reviewed, substantial heterogeneity exists in trial design with respect to patient selection, timing of acute and follow-up scans, GBCA dose, timing of LGE acquisition, and method for MI quantification. Among those RCTs not reporting scanner strength and vendor, GBCA used, and software used for MI size quantification, the majority were multicenter RCTs.

The other major findings were as follows: 1) acute CMR was most commonly performed at 3 to 5 days and follow-up CMR at 6 months; 2) Gd-DOTA, gadobutrol, and Gd-DTPA were most commonly used at a similar dose of 0.20 mmol/kg, with LGE acquired at 10 min; 3) MI size was quantified manually in most RCTs, followed by the 5-SD threshold; 4) STEMI in the left anterior descending coronary artery territory accounted for one-half and those with TIMI flow grades of 0 or 1 accounted for three-quarters of all STEMIs entering these RCTs and could be taken into consideration for sample-size calculation in specific circumstances; 5) for positive RCTs, the most commonly seen effect sizes were 34% for acute MI and 18% for chronic MI (≥1 month) size reduction; 6) using the control arms from RCTs reporting mean MI size, theoretical sample sizes for future clinical cardioprotection studies were provided; and 7) the dropout rate was highest for RCTs performing paired acute and follow-up scans, followed by those performing follow-up CMR only, and smallest in the RCTs performing acute CMR only.

### Robustness of CMR-derived surrogate endpoints

CMR has recently emerged as a robust tool not only for MI size quantification (5) but also to provide additional information on the AAR (to derive MSI) (8), LV ejection fraction (9), and MVO 6, 10 from a single scan. MI size, myocardial salvage, and LV ejection fraction by CMR are highly reproducible (reducing sample size) 8, 9, 11, 12 and are strongly linked to prognosis 5, 7, 13, 14, 15. Furthermore, with recent advances in mapping techniques, multiparametric information can be obtained from native (for the AAR) (16) and post-contrast T1 maps (to derive extracellular volume fraction maps to interrogate the remote myocardium) 17, 18, T2 maps (AAR) (16) and T2* maps (intramyocardial hemorrhage and residual myocardial iron) 19, 20. These additional mapping parameters provide more robust measurement of the edema-based AAR (when compared with angiographic scores) (21), pathophysiological insights in post-STEMI LV remodeling 17, 18, 19, 22, and prognostic information (20). Unlike single-photon emission computed tomography, CMR has superior spatial resolution (14), does not involve radiation, and requires only a single examination, when the patient is relatively stable. Furthermore, MVO and MI size by CMR have been shown to be more prognostic compared with MI size by single-photon emission computed tomography (14). The superior spatial resolution of CMR also allows detection of small MIs that could be missed by relying on wall motion abnormalities on echocardiography alone (23), interrogation of the peri-infarct zone (24), and hypointense core of the MVO (25), which are all prognostic 24, 25, 26. Therefore, it is not surprising that CMR endpoints have gained popularity for use in several RCTs (3).

### Optimal timing of acute CMR post-STEMI

Preclinical studies have shown that performing CMR too early post-reperfusion (day 1) leads to an overestimation of MI size because of a combination of edema and partial volume effect (27). In the clinical setting, acute MI size has also been shown to be dynamic and to decrease significantly in size between days 1 and 7 28, 29 but is stable between days 3 and 4 (8). Recently, Carrick et al. (30) showed that acute MI size was stable between days 1 and 3 and subsequently reduced in size by day 10. Furthermore, late MVO has also been shown to be stable between days 1 and 3 and to reduce in size by day 10 (20), and the persistence of late MVO at 1 week following STEMI was more prognostic (31). The detection of intramyocardial hemorrhage has been shown to peak at day 3, and reduced in size and incidence by day 10, and intramyocardial hemorrhage was more prognostic than MVO (20).

There is no established method to assess the AAR (a pre-requisite to calculate MSI) in the clinical setting by CMR. T2 mapping has recently emerged as more robust than T2-weighted imaging for assessing edema-based AAR (32). Some controversies exist as to whether edema within the AAR follows a bimodal pattern (33) and whether T2-weighted imaging delineates the AAR at all (34) in the preclinical setting. It was previously believed that edema was stable in the first week of a MI (28) using T2-weighted imaging. However, Carrick et al. (30) recently showed that the extent of myocardial edema followed a unimodal pattern and peaked at day 3 in patients.

**Figure 6:**
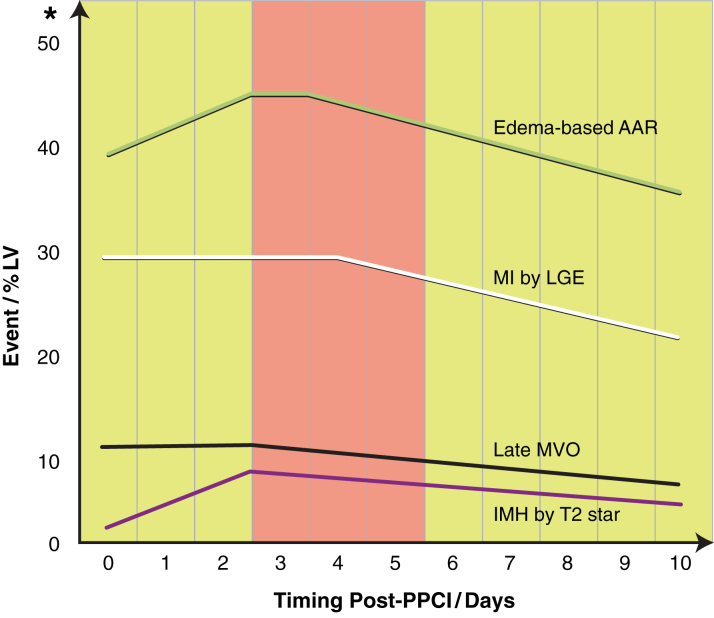
Evolution of Edema-Based Area at Risk, MI Size, Microvascular Obstruction, and Intramyocardial Hemorrhage in Patients With ST-Segment Elevation Myocardial Infarction Within the First 10 Days Post-Reperfusion This is a schematic representation of the evolution edema-based area at risk (AAR) (based on findings from Carrick et al. [30] and Desch et al. [8]), myocardial infarct (MI) size (Carrick et al. [30] and Desch et al. [8]), microvascular obstruction (MVO) (Carrick et al. 20, 30), and intramyocardial hemorrhage (IMH) (Carrick et al. 20, 30) within the first 10 days post-reperfusion, expressed as percentage left ventricular volume or mass (%LV). *The scale on the y-axis is an approximation. LGE = late gadolinium enhancement; PPCI = primary percutaneous coronary intervention.

On the basis of the clinical research published so far, if the CMR scan is performed at <3 days, MI size would be overestimated and AAR would be underestimated. If the CMR scan is performed at >5 days, the edema-based AAR may be underestimated. Therefore, acquiring the acute CMR scan at 3 to 5 days following STEMI, as performed in most cardioprotection RCTs in this review, may be the optimal time to undertake the acute CMR scan, as illustrated in Figure 6.

### Optimal timing of follow-up CMR

Chronic MI size has been shown to be stable when performed between 1 month and up to 1 year (31). Paired acute and follow-up scans also provide information on post-STEMI LV remodeling, which occurs by 2 months, although the process may continue for up to 1 year (31). However, waiting too long between the acute and follow-up CMR scans may increase the likelihood of patients’ dropping out of the studies. Therefore, in RCTs aiming to assess both chronic MI size and LV remodeling, performing the follow-up scan at 6 months, as done in most of the RCTs reviewed in this study, would be optimal.

### Optimal GBCA dose and timing of LGE acquisition

Gadobutrol has been shown to delineate the infarcted myocardium better from the blood pool (better contrast-to-noise ratio) compared with Gd-DTPA (35) and Gd-DOTA (36) in chronic MI. Furthermore, gadobutrol has been shown to differentiate the infarcted myocardium from the LV blood pool as early as 9 min. The recommended relaxivity-adjusted standard doses are 0.22 mmol/kg for Gd-DOTA (36), 0.15 mmol/kg for gadobutrol 35, 36, and 0.20 mmol/kg for Gd-DTPA (35).

Acquiring LGE too early (<8 min) post–GBCA administration has been shown to result in overestimation of MI size (37), and acquiring LGE at 25 min for acute MI size was a better predictor of LV recovery (38). In most RCTs included in this study, LGE imaging was performed at 10 min. Performing comprehensive CMR in patients with STEMI can be time-consuming, and to minimize patient discomfort and prevent dropout of patients, every attempt is made to keep scan time to a minimum. Therefore, acquiring LGE images 15 min post–GBCA administration in future RCTs would be a good compromise.

### Method for quantifying MI size

There is currently no established gold-standard semiautomated technique for MI size quantification. Manual contouring is considered the reference standard 4, 11, but it can be time-consuming and may be subjective. The 5-SD approach is currently recommended, as it may improve reproducibility (4).

Although FWHM has emerged as being the most reproducible 39, 40, it has been shown to underestimate acute and chronic MI size (40). Some studies showed that 5-SD was promising 39, 40, 41, but others showed that it overestimated MI size 42, 43. The n-SD technique requires the remote myocardium to be appropriately nulled and free of artifacts. A manual region of interest is required in the remote myocardium, and this can be a source of variability. The Otsu technique does not require a region of interest as a reference and has been shown to accurately delineate MI size (40). But 2 subsequent studies showed that Otsu overestimated MI size 42, 43.

The most common method used to quantify MI size in the RCTs was manual contouring, followed by 5-SD and FWHM. We recently showed that FWHM underestimates chronic MI size in those with MVO on the acute scan because of very high extracellular volume in the area previously occupied by MVO and should be avoided for RCTs assessing chronic MI size (42).

The 6-SD method appears the most promising and has been shown to have the highest accuracy to predict segment wall recovery in patients with chronic myocardial infarction (44) and is similar to manual quantification in patients with both acute and chronic myocardial infarction 39, 42. The 6-SD approach also performed well when using 2 different LGE sequences (42).

### Impact of patient selection and timing of CMR on sample size

In Table 1 we provide guidance for expected sample sizes, depending on the desired effect size, after accounting for expected dropout rates when planning future RCTs. For example, if an RCT includes patients with all TIMI flow grades pre-PPCI and presenting within 12 h of symptom onset, for an expected 25% reduction in acute MI size, 164 patients would be needed in each arm. If the endpoint were to be changed to chronic MI size, then for the same effect size, the sample size for each arm would increase to 205. However, we have observed that the realistic effect size for chronic MI size on average is lower (18%), and this would increase the sample size further.

If only patients with pre-PPCI TIMI flow grades of 0 or 1 and within 6 h of symptom onset were included, the sample size for each arm would be reduced by about one-third to 110 patients. However, only three-quarters of patients with STEMI present with TIMI flow grades of 0 or 1, and therefore at least one-third more patients would need to be screened to achieve the desired sample size.

### Recommendations for future cardioprotection RCTs in patients with STEMI

Careful patient selection to include those patients most likely benefit from a novel cardioprotective therapy for reducing MI size (ischemic time <6 h and pre-PPCI TIMI flow grade 0 or 1) can reduce sample size by one-third, but more patients would need to be screened.

Acute MI size should be preferred to chronic MI size as a surrogate endpoint, because acute MI size is already prognostic (5), and this would reduce sample size and result in fewer dropouts.

Acute CMR should ideally be performed on day 3, 4, or 5. When a follow-up scan is planned, 6 months would be the optimal timing for data on both chronic MI size and LV remodeling.

The preferred GBCA should be gadobutrol (superior contrast-to-noise ratio) (35) at 0.15 mmol/kg (recommended relaxivity-adjusted standard dose) 35, 36, with LGE performed at 15 min.

Manual quantification of MI size by experienced operators at a core laboratory level is considered the gold standard (4). When this is not practical, the 6-SD threshold would be an alternative option. However, any semiautomated technique is likely to be influenced by the LGE image quality, and therefore, each center may need to validate the performance of these semiautomated techniques at the respective center or core laboratory.

In the absence of a gold-standard method for the AAR, MI size should be reported as %LV, which has also been shown to be prognostic (5). Although MSI is reported to be a better surrogate, in adequately powered RCTs, the AAR should be adequately balanced in both arms, and therefore expressing MI size as %LV would be acceptable.

Finally, all RCTs, in particular multicenter RCTs, should provide adequate details on the execution and quantification of MI size in RCTs to allow fair interpretation and comparison of study results and provide more reliable MI size in the control arms for future sample-size calculation.

### Study limitations

We included only RCTs in this review, but some of the recommendations could also be applied to observational studies to standardize the conduct of CMR, making comparison among studies easier.

We concentrated only on MI size. Using MSI as a surrogate endpoint is considered more sensitive to assess the effectiveness of cardioprotective therapies (45) and has recently been shown to reduce sample size (46). However, given the lack of consensus 33, 34 and ongoing validation of the AAR methodologies, we did not evaluate the use of MSI in the RCTs published so far.

MVO has been shown to be more prognostic than MI size (15), but we did not assess the definition of MVO in the included RCTs. However, if the performance and quantification of LGE for MI size were standardized, this would also standardize MVO (late) quantification.

The recommended dose for gadobutrol is based on the recommended relaxivity-adjusted standard dose, and ideally a head-to-head comparison of the different doses should be performed. However, this is challenging in the acute MI setting, given the dynamic nature of MI size within the first week. The most commonly seen effect sizes for acute and chronic MI size are related to the intervention they were subjected to and are meant to serve as a guide for realistic effect sizes in the acute and chronic MI setting.

## Conclusions

There is significant heterogeneity in the design of RCTs using CMR to quantify MI size in clinical cardioprotection studies in reperfused patients with STEMI. Here, we have provided insights from RCTs published so far and have offered recommendations for standardizing the assessment of MI size by CMR to optimize the design of future studies assessing the efficacy of cardioprotective strategies.

Perspectives **COMPETENCY IN MEDICAL KNOWLEDGE:** Among RCTs using MI size by CMR as a surrogate to assess the effectiveness of cardioprotective strategies, significant heterogeneity exists in the performance of CMR and the analysis of MI size. Furthermore, one-third of the RCTs did not provide details on the CMR scanner used, the contrast agent and dose administered, or the method used to quantify MI size.

**TRANSLATIONAL OUTLOOK:** There is a need to standardize the acquisition of CMR images and the analysis of MI size, to optimize the design of future RCTs, and we have provided some initial recommendations. We have also provided representative MI sizes for patients in the control arms of the RCTs, which could be used for sample-size calculations. Future RCTs should report details on the execution of the scans and the methods used for MI size quantification, to facilitate comparison among RCTs.

## Figures and Tables

**Table 1:** Sample-Size Estimation for Future RCTs Investigating Therapy for Reducing MI Size by CMR

Patient Inclusion Criteria		Pooled MI Size by CMR in the Control Group From Previous RCTs (as %LV)			Sample Size per Group After Accounting for Potential Dropouts (9% for Acute CMR and 13% for Follow-Up CMR) for 90% Power With a 2-Sided Alpha of 0.05					
					For an Effect Size of 20%		For an Effect Size of 25%		For an Effect Size of 30%	
TIMI Flow Pre-PPCI	Symptom Onset (h)	Weighted Mean (95% CI)	SD	Number of Studies (Number of Patients)	MI Size	Sample Size	MI Size	Sample Size	MI Size	Sample Size
Acute scan only (including STEMI in all coronary territories)										
0 or 1	≤6	22 (20–24)	12	3 RCTs (136 patients): Chan (2012) Garcia-Dorado (2014) Waltenberg (2014)	17.6	171	16.5	110	15.4	76
0 or 1	≤12	24 (22–26)	13	3 RCTs (189 patients): Freixa (2011) Mewton (2013) Siddiqi (2014) White (2015)	19.2	169	18.0	108	16.8	75
0–3	≤12	21 (19–22)	14	5 RCTs (305 patients): Yoon (2013) Ko (2014) Hoole (2015) McCann (2015) Liu (2016)	16.8	265	15.8	164	14.7	85
Chronic scan only (including STEMI in all coronary territories)										
0 or 1	≤6	15 (14–17)	9	4 RCTs (152 patients): Tarantini (2012) Atar (2015) Waltenberg (2014) Roos (2016)	12.0	215	11.3	215	10.5	96
0 or 1	≤12	15 (13–16)	9	4 RCTs (186 patients): Lonborg (2010) Freixa (2011) Lonborg (2012) Siddiqi (2014)	12.0	215	11.3	216	10.5	96
0–3	≤12	15 (14–17)	11	4 RCTs (284 patients): Song (2009) Ranchord (2012) Roolvink (2016) Ko (2014)	12.0	320	11.3	205	10.5	142

The references for the RCTs included in each row are provided in Online Table 5.

CI = confidence interval; CMR = cardiac magnetic resonance; MI = myocardial infarction; %LV = percentage of left ventricular mass or volume; PPCI = primary percutaneous coronary intervention; RCT = randomized controlled trial; TIMI = Thrombolysis in Myocardial Infarction.
