# Supplementary material for: Myocardial Infarct Size by CMR in Clinical Cardioprotection Studies: Insights From Randomized Controlled Trials
Source: JACC Cardiovasc Imaging. 2017 Mar;10(3):230–40. doi: 10.1016/j.jcmg.2017.01.008 (PMC5348096; doi:10.1016/j.jcmg.2017.01.008)
Supplement: Online Data [file mmc1.docx]

**Online Appendix**

**Legend**

**Methods: study selection and screening**

**Table 1: RCTs included in this study**

**Table 2: Gadolinium based contrast agents used in RCTs**

**Table 3: RCTs quantifying acute MI size and arranged in order of duration of symptoms, vessels included and TIMI flow pre-PPCI**

**Table 4: RCTs quantifying chronic MI size by CMR**

**Methods: study selection and screening**

We performed a comprehensive literature search in the MEDLINE and EMBASE electronic databases via OVID up to November 23, 2016. *The search terms used were:*

- cardiovascular
- cardiovascular magnetic resonance
- controlled
- elevation
- imaging
- infarction
- magnetic
- magnetic resonance imaging
- myocardial
- myocardial infarction
- randomized
- randomized controlled trial
- resonance
- st-segment
- st-segment elevation myocardial infarction

trial

The study screening and selection as per the preferred reporting items for systematic reviews and meta-analyses (PRISMA).[1] A total of 399 articles were identified and after screening the abstracts, the full text of 89 articles were further scrutinized for inclusion in this study. 62 RCTs involving 10,570 patients met the inclusion criteria.

**Table 1: RCTs included in this study**

| **Study** | **No of patients** | **Treatment strategy in intervention arm** | **CMR Outcome** |
| --- | --- | --- | --- |
| **Lunde 2006[2]** | 100 | IC Bone marrow cells | No difference in MI size |
| **Thiele 2008[3] / Eitel 2011[4]** | 154 | IC vs IV Abciximab | 34% reduction in acute MI size |
| **Atar 2009[5]** | 234 | IV FX06 | No difference in MI size |
| **Sardella 2009[6]** | 175 | Thrombectomy | 18% reduction in chronic MI size |
| **Song 2009** **[7]** | 39 | IV Tirofiban | No difference in MI size |
| **Bertrand 2010** **[8]** | 105 | Abciximab IC versus IV, low dose versus high dose (2X2) | No difference in MI size |
| **Desmet 2010** **[9]** | 110 | IC Adenosine | No difference in MSI or MVO |
| **Götberg 2010** **[10]** | 20 | Hypothermia | 34% reduction in acute MI size |
| **Haeck 2010** **[11]** | 206 | Proximal embolic protection device and thrombus aspiration | No difference in MI size |
| **Lønborg 2010** **[12]** | 118 | Ischemic Postconditioning | 18% reduction in chronic MI size |
| **Patel 2010** **[13]** | 99 | IV Pexelizumab | 35% reduction in acute MI size |
| **Sorensson 2010[14]/ 2013** **[15]** | 76 | Ischemic Postconditioning | No difference in MI size as a % of AAR |
| **Suh 2010** **[16]** | 57 | IV Erythropoeitin | No difference in MI size |
| **Thiele 2010** **[17]** | 251 | IV N-acetylcysteine | No difference in MSI, MI size |
| **Freixa 2011[18]** | 79 | Ischemic Postconditioning | No difference in MI size |
| **Hirsch 2011** **[19]** | 200 | IC Bone marrow cells or peripheral mononuclear cells | No difference in MI size |
| **Ludman 2011**[20] | 51 | IV Erythropoeitin | No difference in MI size |
| **Najjar 2011[21]** | 222 | IV Erythropoeitin | No difference in MI size |
| **Patel 2011[22]** | 337 | Intra-aortic balloon pump | No difference in MI size |
| **Thiele 2011[23]** | 158 | IV Tenectaplase | No difference in MI size |
| **Chan 2012** **[24]** | 60 | IV Deferoxamine | No difference in MI size |
| **Lønborg 2012** **[25]** | 148 | IV Exenatide | 15% improvement in MSI, no difference in MI size as a percentage of the LV |
| **Prunier 2012** **[26]** | 110 | IV Erythropoeitin | No difference in MI size |
| **Ranchord 2012** **[27]** | 136 | Oxygen | No difference in MI size |
| **Song 2012** **[28]** | 198 | High dose Clopidogrel | 20% reduction in acute MI size |
| **Stone 2012** **[29]** | 452 | IV Abciximab with or without aspiration (2X2 factorial design) | 16% reduction in chronic MI size |
| **Tarantini 2012** **[30]** | 78 | Ischemic Postconditioning | No difference in MI size |
| **Thuny 2012** **[31]** | 50 | Ischemic Postconditioning | 38% reduction in acute MI size |
| **Wohrle 2012** **[32]** | 51 | IV Bivalirudin vs Heparin + Abciximab | No difference in MI size |
| **Crimi 2013** **[33]** | 100 | Remote ischemic postconditioning - lower limb | No difference in MI size |
| **Dwyer 2013** **[34]** | 102 | Ischemic Postconditioning | No difference in MSI or MI size |
| **Eitel 2013** **[35]** | 795 | IC vs IV Abciximab | No difference in MI size |
| **Ibanez 2013[36]/** **Pizarro 2014** **[37]** | 270 | IV Metoprolol | 16% reduction in acute MI size, 14% reduction in chronic MI size |
| **Mewton 2013** **[38]** | 50 | Ischemic Postconditioning | 35% reduction in acute MI size |
| **Woo 2013** **[39]** | 58 | IV Exenatide | 46% reduction in chronic MI size |
| **Yoon 2013** **[40]** | 126 | Distal protection device | MVO increased by treatment strategy |
| **Garcia-Dorado 2014** **[41]** | 201 | IC Adenosine | No difference in MI size |
| **Jones 2014** **[42]** | 80 | IC Nitrite | No difference in MI size |
| **Ko 2014** **[43]** | 185 | Rosuvastatin | No difference in MI size |
| **Limalanathan 2014** **[44]** | 272 | Ischemic Postconditioning | No difference in MI size |
| **Siddiqi 2014** **[45]** | 229 | IV Nitrite | No difference in MI size |
| **Waltenberger 2014** **[46]** | 52 | Pacing post-conditioning | 29% reduction in acute MI size, 25% reduction in chronic MI size |
| **Carrick 2014** **[47]** | 101 | Deferred PCI after initial coronary reperfusion | 21% improvement in MSI |
| **Roubille 2014** **[48]** | 91 | Ischemic Postconditioning | No difference in MI size |
| **Erlinge 2014** **[49]** | 120 | Hypothermia | No difference in MI size/AAR |
| **Atar 2015** **[50]** | 163 | IV TRO40303 | No difference in MI size |
| **White 2015** **[51]** | 197 | Remote ischaemic perconditioning - upper limb | 27% reduction in acute MI size |
| **Kim 2015** **[52]** | 111 | Ischemic Postconditioning | No difference in MSI |
| **Nichol 2015** **[53]** | 46 | Intraperitoneal hypothermia | No difference in chronic MI size |
| **Stub 2015** **[54]** | 470 | Oxygen | No difference in MI size |
| **Hoole 2015** **[55]** | 41 | Manual thrombectomy versus balloon angioplasty | No difference in MVO and MI size |
| **Kim 2015** **[56]** | 67 | High dose atorvastatin | No difference in MI size/AAR |
| **McCann 2015** **[57]** | 205 | Complete in-hospital revascularisation | No difference in MI size |
| **Deftereos 2015** **[58]** | 151 | Colchicine | 35% reduction in acute MI size |
| **Eitel 2015** **[59]** | 696 | Remote ischaemic perconditioning - upper limb (RIC) + Ischemic Postconditioning vs Ischemic Postconditioning vs control | 23% improvement in MSI with RIC+PostC |
| **Nazir 2016** **[60]** | 247 | Adenosine versus Sodium Nitroprusside vs placebo | No difference in MSI or MI size |
| **Roolvink 2016** **[61]** | 683 | IV Metoprolol | No difference in MI size |
| **Roos 2016** **[62]** | 199 | IV exenatide | No difference in MI size |
| **Belle 2016[63]** | 140 | Deferred stenting | A trend towards more MVO in the deferred stenting arm |
| **Liu 2016 [64]** | 119 | Remote ischemic conditioning – upper limb | Small MVO and MI size in the intervention arm |
| **Dominguez-Rodriguez 2016[65]** | 146 | IV and IC melatonin | No difference in MI size |
| **Verouhis 2016[66]** | 93 | Remote ischemic conditioning – lower limb | No difference in MI size |

CMR: cardiovascular magnetic resonance; MI: myocardial infarct; IC: intracoronary; IV: intravenous; MSI: myocardial salvage index; MVO: microvascular obstruction

**Table 2: Gadolinium based contrast agents used in RCTs**

| **GBCAs** | **Dose/ mmol/kg** | **Time of LGE acquisition post-contrast (mins)** | **Number of RCTs** |
| --- | --- | --- | --- |
| Gd-DOTA (Dotarem) | 0.10 | 10 | 1 |
|  | 0.15 | 15 | 1 |
|  | 0.20 | 8 | 1 |
|  | 0.20 | 10 | 7 |
|  | 0.20 | 15 | 2 |
| Gadobutrol  (Gadovist) | 0.10 | 10 | 1 |
|  | 0.15 | 10 | 1 |
|  | 0.15 | 15 | 1 |
|  | 0.20 | 5 | 1 |
|  | 0.20 | 10 | 2 |
|  | 0.10 | Not specified | 1 |
|  | 0.20 | Not specified | 1 |
|  | 0.15 to 0.20 | 10 | 1 |
| Gd-DTPA  (Magnevist) | 0.15 | 10 | 2 |
|  | 0.15 | 15 | 2 |
|  | 0.15 | Not specified | 1 |
|  | 0.20 | 10 | 8 |
|  | 0.20 | 15 | 4 |
|  | 0.20 | Not specified | 1 |
| Gd-BOPTA  (MultiHance) | 0.10 | 15 | 1 |
| Gadodiamide  (Omniscan) | 0.20 | 10 | 1 |
|  | 0.20 | 15 | 1 |
|  | 0.25 | 20 | 1 |
| GBCA: gadolinium-based contrast agent; LGE: late gadolinium enhancement; RCTs: randomized controlled trials | | | |

**Table 3: RCTs quantifying acute MI size and arranged in order of duration of symptoms, vessels included and TIMI flow pre-PPCI**

| **Study** | **Vessels** | **Symptom duration** | **Timing of CMR** | **TIMI pre-PPCI** | **Scanner strength** | **GBCA dose/ mmol/kg** | **GBCA** | **Timing of LGE/ mins** | **Method of MI quantification** | **Number in control arm** | **MI size/ %LV mass** | **SD** |
| --- | --- | --- | --- | --- | --- | --- | --- | --- | --- | --- | --- | --- |
| **≤6 hours** | | | | | | | | | | | | |
| **Garcia-Dorado 2014** **[41]** | All | 6 | 2 to 7 days | 0-1 | 1.5T | 0.2 | Magnevist | 10 | 5 SD | 86 | **22.5** | **14.0** |
| **Waltenberg 2014** **[46]** | All | 6 | 4 days | 0-1 | Not specified | 0.2 | Magnevist | 10 | 5 SD | 27 | **21.7** | **2.3** |
| **Chan 2012[24]** | All | 6 | 2 to 4 days | 0-3 | 1.5T | 0.2 | Magnevist | 15 | Manual | 23 | **18.6** | **10.2** |
| **Götberg 2010[10]** | LAD and RCA | 6 | 2 to 6 days | 0-3 | 1.5T | 0.2 | Dotarem | 15 | Automated | 9 | **20.5** | **10.0** |
| **Crimi 2013[33]** | LAD | 6 | 3 to 5 days | 0-1 | 1.5T | 0.2 | Magnevist | 15 | Manual | 42 | **26.0** | **13.0** |
| **Ibanez 2013[36]** | LAD | 6 | 5 to 7 days | 0-3 | 1.5 and 3T | 0.2 | Magnevist | 10 | Manual | 114 | **25.1** | **13.9** |
| **6-12 hours** | | | | | | | | | | | | |
| **Najjar 2011** **[21]** | All | 8 | 2 to 6 days | 0-1 | 1.5T | Not specified | Not specified | Not specified | Manual | 68 | **15.0** | **10.0** |
| **Sardella 2009[6]** | All | 9 | 3 to 5 days | 0-1 | 1.5T | 0.1 | MultiHance | 15 | Manual | 37 | **13.0** | **6.7** |
| **≤12 hours** | | | | | | | | | | | | |
| **Siddiqi 2014[45]** | All | 12 | 6 to 8 days | 0-1 | Not specified | Not specified | Not specified | Not specified | Manual | 88 | **23.1** | **13.2** |
| **Freixa 2011[45]** | All | 12 | 7 days | 0-1 | 1.5T | 0.2 | Omniscan | 10 | 2 SD | 36 | **22.1** | **10.2** |
| **Mewton 2013[45]** | All | 12 | 7 days | 0-1 | 1.5T | 0.2 | Dotarem | 10 | Manual | 25 | **27.6** | **16.0** |
| **Liu 2016[64]** | ALL | 12 | 3-7 days | 0-3 | 1.5T | 0.1 | Gadovist | Not specified | Manual | 60 | **16.6** | **6.7** |
| **Hoole 2015[55]** | All | 12 | 2 days | 0-3 | 1.5T | 0.2 | Gadovist | 5 | Not specified | 15 | **14.4** | **7.7** |
| **McCann 2015[57]** | All | 12 | 3 days | 0-3 | 1.5T | 0.2 | Magnevist | 10 | FWHM | 105 | **15.9** | **13.2** |
| **Yoon 2013[40]** | All | 12 | 3 to 5 days | 0-3 | 1.5T | 0.2 | Dotarem | 8 | 5 SD | 55 | **35.7** | **14.6** |
| **Ko 2014[43]** | All | 12 | 3 to 7 days | 0-3 | 1.5T | 0.2 | Not specified | 10 | Manual | 70 | **20.5** | **11.7** |
| **White 2015[51]** | All | 12 | 3 to 6 days | 0 | 1.5T | 0.1 | Dotarem | 10 | Otsu | 40 | **24.5** | **12** |
| **Suh 2010[16]** | LAD - proximal or mid | 12 | 4 days | 0 | 1.5T | 0.2 | Magnevist | 10 | Not specified | 25 | **37.0** | **13.8** |
| **Lunde 2006[2]** | LAD | 12 | 2-3 weeks | 0-3 | 1.5T | 0.2 | Magnevist | 10 | Manual | 44 | **22.2** | **14.0** |

**Table 4: RCTs quantifying chronic MI size by CMR**

| **Study** | **Vessels** | **Symptom duration** | **Timing of CMR post-PPCI** | **TIMI pre-PPCI** | **Scanner strength** | **GBCA** | **GBCA dose (mmol/kg)** | **Timing of LGE/ mins** | **Method of MI quantification** | **Number in control arm** | **MI size/ %LV** | **SD** |
| --- | --- | --- | --- | --- | --- | --- | --- | --- | --- | --- | --- | --- |
| **Atar 2015** **[50]** | All | 6 | 1 month | 0-1 | 1.5T | not specified | 0.2 | 15 | Not specified | 43 | **15.0** | **10.0** |
| **Woo 2013** **[39]** | All | 12 | 1 month | 0 | 3T | Gadovist | 0.2 | NA | 2 SD | 40 | **21.9** | **10.4** |
| **Tarantini 2012** **[30]** | All | 6 | 1 month | 0-1 | 1.5T | not specified | 0.1 | 10 to 15 | 5 SD | 39 | **14.3** | **9.9** |
| **Song 2009[7]** | All | 12 | 1 month | 0-3 | 1.5T | Magnevist | 0.15 | 10 to 15 | Manual | 20 | **25.2** | **13.2** |
| **Ranchord 2012[27]** | All | 12 | 1 month | 0-3 | 1.5T | Magnevist | 0.2 | 15 | Not specified | 28 | **13.1** | **9.7** |
| **Roolvink 2016** **[61]** | All | 12 | 1 month | 0-3 | Not specified | Not specified | Not specified | Not specified | Not specified | 169 | **14.1** | **11.5** |
| **Najjar 2011[21]** | All | 8 | 3 months | 0-1 | 1.5T | Not specified | Not specified | Not specified | Manual | 63 | **10.4** | **7.6** |
| **Sardella 2009[6]** | All | 9 | 3 months | 0-1 | 1.5T | MultiHance | 0.1 | 15 | Manual | 36 | **11.0** | **8.7** |
| **Lønborg 2010[12]** | All | 12 | 3 months | 0-1 | 1.5T | Magnevist | 0.2 | not specified | Not specified | 43 | **17.0** | **8.0** |
| **Lønborg 2012** **[25]** | All | 12 | 3 months | 0-1 | 1.5T | Gadovist | 0.1 | 10 | Automated | 57 | **12.0** | **6.0** |
| **Ko 2014** **[43]** | All | 12 | 3 months | 0-3 | 1.5T | not specified | 0.2 | 10 to 15 | Manual | 67 | **15.8** | **9.7** |
| **Waltenberger 2014[46]** | All | 6 | 4 months | 0-1 | Not specified | Magnevist | 0.2 | 10 | 5 SD | 26 | **18.6** | **2.0** |
| **Roos 2016 [62]** | All | 6 | 4 months | 0-1 | Not specified | Not specified | Not specified | Not specified | Not specified | 44 | **15.1** | **10.5** |
| **Crimi 2013[33]** | LAD | 6 | 4 months | 0-1 | 1.5T | Magnevist | 0.2 | 15 | Manual | 36 | **20.0** | **9.0** |
| **Pizarro 2014[37]** | LAD | 6 | 6 months | 0-3 | 1.5T and 3T | Magnevist | 0.2 | 10 to 15 | Manual | 101 | **18.3** | **9.8** |
| **Freixa 2011[18]** | All | 12 | 6 months | 0-1 | 1.5T | Omniscan | 0.2 | 10 to 15 | 2 SD | 31 | **18.7** | **10.6** |
| **Siddiqi 2014[45]** | All | 12 | 6 months | 0-1 | Not specified | Not specified | Not specified | Not specified | Manual | 55 | **15.0** | **9.7** |
| **Lunde 2006[2]** | LAD | 12 | 6 months | 0-3 | 1.5T | Magnevist | 0.2 | 10 to 20 | Manual | 47 | **19.6** | **12.5** |

CMR: cardiovascular magnetic resonance; TIMI: thrombolysis in myocardial infarction; PPCI: primary percutaneous coronary intervention; GBCA: gadolinium-based contrast agent; LGE: late gadolinium enhancement; LV: left ventricle; MI: myocardial infarct; CI: confidence interval; SD: standard deviation

**Table 5: Sample size estimation for future RCTs investigating therapy for reducing MI size by CMR**

| **Patient inclusion criteria** | | **Pooled MI size by CMR in the control group from previous RCTs (as % of LV mass or volume)** | | | **Sample size per group after accounting for potential dropouts (9% for acute CMR and 13% for follow-up CMR) for 90% power and with a 2-sided alpha of 0.05** | | | | | |
| --- | --- | --- | --- | --- | --- | --- | --- | --- | --- | --- |
| **TIMI flow pre-PPCI** | **Symptom onset (hours)** | **Weighted mean**  **(95% CI)** | **SD** | **No. of Studies**  **(No. of patients)** | **For an effect size of 20%** | | **For an effect size of 25%** | | **For an effect size of 30%** | |
|  |  |  |  |  | **MI size** | **Sample size** | **MI size** | **Sample size** | **MI size** | **Sample size** |
| **Acute Scan only (including STEMI in all coronary territories)** | | | | | | | | | | |
| 0-1 | ≤ 6 | 22 (20 to 24) | 12 | 3 RCTs  (136 patients)  Chan 2012[24]  Garcia-Dorado 2014[41]  Waltenberg 2014[46] | 17.6 | 171 | 16.5 | 110 | 15.4 | 76 |
| 0-1 | ≤ 12 | 24 (22 to 26) | 13 | 3 RCTs  (189 patients)  Freixa 2011[18]  Mewton 2013[38]  Siddiqi 2014[45]  White 2015[51] | 19.2 | 169 | 18.0 | 108 | 16.8 | 75 |
| 0-3 | ≤ 12 | 21 (19 to 22) | 14 | 5 RCTs  (305 patients)  Yoon 2013[40]  Ko 2014[43]  Hoole 2015[55]  McCann 2015[57]  Liu 2016[64] | 16.8 | 265 | 15.8 | 164 | 14.7 | 85 |
| Chronic Scan only (including STEMI in all coronary territories) | | | | | | | | | | |
| 0-1 | ≤ 6 | 15 (14 to 17) | 9 | 4  (152 patients)  Tarantini 2012[30]  Atar 2015[50]  Waltenberg 2014[46]  Roos 2016[62] | 12.0 | 215 | 11.3 | 215 | 10.5 | 96 |
| 0-1 | ≤ 12 | 15 (13 to 16) | 9 | 4 RCTs  (186 patients)  Lonborg 2010[12]  Freixa 2011[18]  Lonborg 2012 [25]  Siddiqi 2014[45] | 12.0 | 215 | 11.3 | 216 | 10.5 | 96 |
| 0-3 | ≤ 12 | 15 (14 to 17) | 11 | 4 RCTs  (284 patients)  Song 2009[7]  Ranchord 2012[27]  Roolvink 2016[61]  Ko 2014[43] | 12.0 | 320 | 11.3 | 205 | 10.5 | 142 |

RCT: randomized controlled trial; CMR: cardiovascular magnetic resonance; LV: left ventricle; MI: myocardial infarct; CI: confidence interval; TIMI: thrombolysis in myocardial infarction; PPCI: primary percutaneous coronary intervention; SD: standard deviation

1. Moher D, Liberati A, Tetzlaff J, Altman DG, Group P. Preferred reporting items for systematic reviews and meta-analyses: the PRISMA statement. Bmj. 2009;339:b2535. doi:10.1136/bmj.b2535.

2. Lunde K, Solheim S, Aakhus S, Arnesen H, Abdelnoor M, Egeland T et al. Intracoronary injection of mononuclear bone marrow cells in acute myocardial infarction. The New England journal of medicine. 2006;355(12):1199-209. doi:10.1056/NEJMoa055706.

3. Thiele H, Schindler K, Friedenberger J, Eitel I, Furnau G, Grebe E et al. Intracoronary compared with intravenous bolus abciximab application in patients with ST-elevation myocardial infarction undergoing primary percutaneous coronary intervention: the randomized Leipzig immediate percutaneous coronary intervention abciximab IV versus IC in ST-elevation myocardial infarction trial. Circulation. 2008;118(1):49-57. doi:10.1161/CIRCULATIONAHA.107.747642.

4. Eitel I, Friedenberger J, Fuernau G, Dumjahn A, Desch S, Schuler G et al. Intracoronary versus intravenous bolus abciximab application in patients with ST-elevation myocardial infarction undergoing primary percutaneous coronary intervention: 6-month effects on infarct size and left ventricular function. The randomised Leipzig Immediate PercutaneouS Coronary Intervention Abciximab i.v. versus i.c. in ST-Elevation Myocardial Infarction Trial (LIPSIAbciximab-STEMI). Clinical research in cardiology : official journal of the German Cardiac Society. 2011;100(5):425-32. doi:10.1007/s00392-010-0260-5.

5. Atar D, Petzelbauer P, Schwitter J, Huber K, Rensing B, Kasprzak JD et al. Effect of intravenous FX06 as an adjunct to primary percutaneous coronary intervention for acute ST-segment elevation myocardial infarction results of the F.I.R.E. (Efficacy of FX06 in the Prevention of Myocardial Reperfusion Injury) trial. Journal of the American College of Cardiology. 2009;53(8):720-9. doi:10.1016/j.jacc.2008.12.017.

6. Sardella G, Mancone M, Bucciarelli-Ducci C, Agati L, Scardala R, Carbone I et al. Thrombus aspiration during primary percutaneous coronary intervention improves myocardial reperfusion and reduces infarct size: the EXPIRA (thrombectomy with export catheter in infarct-related artery during primary percutaneous coronary intervention) prospective, randomized trial. Journal of the American College of Cardiology. 2009;53(4):309-15. doi:10.1016/j.jacc.2008.10.017.

7. Song YB, Hahn JY, Gwon HC, Kim JH, Lee SY, Choe YH et al. Upstream high-dose tirofiban does not reduce myocardial infarct size in patients undergoing primary percutaneous coronary intervention: a magnetic resonance imaging pilot study. Clinical cardiology. 2009;32(6):321-6. doi:10.1002/clc.20551.

8. Bertrand OF, Rodes-Cabau J, Larose E, Rinfret S, Gaudreault V, Proulx G et al. Intracoronary compared to intravenous Abciximab and high-dose bolus compared to standard dose in patients with ST-segment elevation myocardial infarction undergoing transradial primary percutaneous coronary intervention: a two-by-two factorial placebo-controlled randomized study. The American journal of cardiology. 2010;105(11):1520-7. doi:10.1016/j.amjcard.2010.01.006.

9. Desmet W, Bogaert J, Dubois C, Sinnaeve P, Adriaenssens T, Pappas C et al. High-dose intracoronary adenosine for myocardial salvage in patients with acute ST-segment elevation myocardial infarction. European heart journal. 2011;32(7):867-77. doi:10.1093/eurheartj/ehq492.

10. Gotberg M, Olivecrona GK, Koul S, Carlsson M, Engblom H, Ugander M et al. A pilot study of rapid cooling by cold saline and endovascular cooling before reperfusion in patients with ST-elevation myocardial infarction. Circulation Cardiovascular interventions. 2010;3(5):400-7. doi:10.1161/CIRCINTERVENTIONS.110.957902.

11. Haeck JD, Kuijt WJ, Koch KT, Bilodeau L, Henriques JP, Rohling WJ et al. Infarct size and left ventricular function in the PRoximal Embolic Protection in Acute myocardial infarction and Resolution of ST-segment Elevation (PREPARE) trial: ancillary cardiovascular magnetic resonance study. Heart. 2010;96(3):190-5. doi:10.1136/hrt.2009.180448.

12. Lonborg J, Kelbaek H, Vejlstrup N, Jorgensen E, Helqvist S, Saunamaki K et al. Cardioprotective effects of ischemic postconditioning in patients treated with primary percutaneous coronary intervention, evaluated by magnetic resonance. Circulation Cardiovascular interventions. 2010;3(1):34-41. doi:10.1161/CIRCINTERVENTIONS.109.905521.

13. Patel MR, Worthley SG, Stebbins A, Dill T, Rademakers FE, Valeti US et al. Pexelizumab and infarct size in patients with acute myocardial infarction undergoing primary percutaneous coronary Intervention: a delayed enhancement cardiac magnetic resonance substudy from the APEX-AMI trial. JACC Cardiovascular imaging. 2010;3(1):52-60. doi:10.1016/j.jcmg.2009.09.014.

14. Sorensson P, Saleh N, Bouvier F, Bohm F, Settergren M, Caidahl K et al. Effect of postconditioning on infarct size in patients with ST elevation myocardial infarction. Heart. 2010;96(21):1710-5. doi:10.1136/hrt.2010.199430.

15. Sorensson P, Ryden L, Saleh N, Tornvall P, Arheden H, Pernow J. Long-term impact of postconditioning on infarct size and left ventricular ejection fraction in patients with ST-elevation myocardial infarction. BMC cardiovascular disorders. 2013;13:22. doi:10.1186/1471-2261-13-22.

16. Suh JW, Chung WY, Kim YS, Kim KI, Jeon EJ, Cho YS et al. The effect of intravenous administration of erythropoietin on the infarct size in primary percutaneous coronary intervention. International journal of cardiology. 2011;149(2):216-20. doi:10.1016/j.ijcard.2010.02.002.

17. Thiele H, Hildebrand L, Schirdewahn C, Eitel I, Adams V, Fuernau G et al. Impact of high-dose N-acetylcysteine versus placebo on contrast-induced nephropathy and myocardial reperfusion injury in unselected patients with ST-segment elevation myocardial infarction undergoing primary percutaneous coronary intervention. The LIPSIA-N-ACC (Prospective, Single-Blind, Placebo-Controlled, Randomized Leipzig Immediate PercutaneouS Coronary Intervention Acute Myocardial Infarction N-ACC) Trial. Journal of the American College of Cardiology. 2010;55(20):2201-9. doi:10.1016/j.jacc.2009.08.091.

18. Freixa X, Bellera N, Ortiz-Perez JT, Jimenez M, Pare C, Bosch X et al. Ischaemic postconditioning revisited: lack of effects on infarct size following primary percutaneous coronary intervention. European heart journal. 2012;33(1):103-12. doi:10.1093/eurheartj/ehr297.

19. Hirsch A, Nijveldt R, van der Vleuten PA, Tijssen JG, van der Giessen WJ, Tio RA et al. Intracoronary infusion of mononuclear cells from bone marrow or peripheral blood compared with standard therapy in patients after acute myocardial infarction treated by primary percutaneous coronary intervention: results of the randomized controlled HEBE trial. European heart journal. 2011;32(14):1736-47. doi:10.1093/eurheartj/ehq449.

20. Ludman AJ, Yellon DM, Hasleton J, Ariti C, Babu GG, Boston-Griffiths E et al. Effect of erythropoietin as an adjunct to primary percutaneous coronary intervention: a randomised controlled clinical trial. Heart. 2011;97(19):1560-5. doi:10.1136/hrt.2011.223867.

21. Najjar SS, Rao SV, Melloni C, Raman SV, Povsic TJ, Melton L et al. Intravenous erythropoietin in patients with ST-segment elevation myocardial infarction: REVEAL: a randomized controlled trial. JAMA : the journal of the American Medical Association. 2011;305(18):1863-72. doi:10.1001/jama.2011.592.

22. Patel MR, Smalling RW, Thiele H, Barnhart HX, Zhou Y, Chandra P et al. Intra-aortic balloon counterpulsation and infarct size in patients with acute anterior myocardial infarction without shock: the CRISP AMI randomized trial. JAMA : the journal of the American Medical Association. 2011;306(12):1329-37. doi:10.1001/jama.2011.1280.

23. Thiele H, Eitel I, Meinberg C, Desch S, Leuschner A, Pfeiffer D et al. Randomized comparison of pre-hospital-initiated facilitated percutaneous coronary intervention versus primary percutaneous coronary intervention in acute myocardial infarction very early after symptom onset: the LIPSIA-STEMI trial (Leipzig immediate prehospital facilitated angioplasty in ST-segment myocardial infarction). JACC Cardiovascular interventions. 2011;4(6):605-14. doi:10.1016/j.jcin.2011.01.013.

24. Chan W, Taylor AJ, Ellims AH, Lefkovits L, Wong C, Kingwell BA et al. Effect of iron chelation on myocardial infarct size and oxidative stress in ST-elevation-myocardial infarction. Circulation Cardiovascular interventions. 2012;5(2):270-8. doi:10.1161/CIRCINTERVENTIONS.111.966226.

25. Lonborg J, Vejlstrup N, Kelbaek H, Botker HE, Kim WY, Mathiasen AB et al. Exenatide reduces reperfusion injury in patients with ST-segment elevation myocardial infarction. European heart journal. 2012;33(12):1491-9. doi:10.1093/eurheartj/ehr309.

26. Prunier F, Biere L, Gilard M, Boschat J, Mouquet F, Bauchart JJ et al. Single high-dose erythropoietin administration immediately after reperfusion in patients with ST-segment elevation myocardial infarction: results of the erythropoietin in myocardial infarction trial. Am Heart J. 2012;163(2):200-7 e1. doi:10.1016/j.ahj.2011.11.005.

27. Ranchord AM, Argyle R, Beynon R, Perrin K, Sharma V, Weatherall M et al. High-concentration versus titrated oxygen therapy in ST-elevation myocardial infarction: a pilot randomized controlled trial. Am Heart J. 2012;163(2):168-75. doi:10.1016/j.ahj.2011.10.013.

28. Song YB, Hahn JY, Gwon HC, Chang SA, Lee SC, Choe YH et al. A high loading dose of clopidogrel reduces myocardial infarct size in patients undergoing primary percutaneous coronary intervention: a magnetic resonance imaging study. Am Heart J. 2012;163(3):500-7. doi:10.1016/j.ahj.2011.12.007.

29. Stone GW, Maehara A, Witzenbichler B, Godlewski J, Parise H, Dambrink JH et al. Intracoronary abciximab and aspiration thrombectomy in patients with large anterior myocardial infarction: the INFUSE-AMI randomized trial. JAMA : the journal of the American Medical Association. 2012;307(17):1817-26. doi:10.1001/jama.2012.421.

30. Tarantini G, Favaretto E, Marra MP, Frigo AC, Napodano M, Cacciavillani L et al. Postconditioning during coronary angioplasty in acute myocardial infarction: the POST-AMI trial. International journal of cardiology. 2012;162(1):33-8. doi:10.1016/j.ijcard.2012.03.136.

31. Thuny F, Lairez O, Roubille F, Mewton N, Rioufol G, Sportouch C et al. Post-conditioning reduces infarct size and edema in patients with ST-segment elevation myocardial infarction. Journal of the American College of Cardiology. 2012;59(24):2175-81. doi:10.1016/j.jacc.2012.03.026.

32. Wohrle J, Merkle N, Kunze M, Cristea E, Mehran R, Rottbauer W et al. Effect of bivalirudin compared with unfractionated heparin plus abciximab on infarct size and myocardial recovery after primary percutaneous coronary intervention: the horizons-AMI CMRI substudy. Catheterization and cardiovascular interventions : official journal of the Society for Cardiac Angiography & Interventions. 2012;79(7):1083-9. doi:10.1002/ccd.23179.

33. Crimi G, Pica S, Raineri C, Bramucci E, De Ferrari GM, Klersy C et al. Remote ischemic post-conditioning of the lower limb during primary percutaneous coronary intervention safely reduces enzymatic infarct size in anterior myocardial infarction: a randomized controlled trial. JACC Cardiovascular interventions. 2013;6(10):1055-63. doi:10.1016/j.jcin.2013.05.011.

34. Dwyer NB, Mikami Y, Hilland D, Aljizeeri A, Friedrich MG, Traboulsi M et al. No cardioprotective benefit of ischemic postconditioning in patients with ST-segment elevation myocardial infarction. Journal of interventional cardiology. 2013;26(5):482-90. doi:10.1111/joic.12064.

35. Eitel I, Wohrle J, Suenkel H, Meissner J, Kerber S, Lauer B et al. Intracoronary compared with intravenous bolus abciximab application during primary percutaneous coronary intervention in ST-segment elevation myocardial infarction: cardiac magnetic resonance substudy of the AIDA STEMI trial. Journal of the American College of Cardiology. 2013;61(13):1447-54. doi:10.1016/j.jacc.2013.01.048.

36. Ibanez B, Macaya C, Sanchez-Brunete V, Pizarro G, Fernandez-Friera L, Mateos A et al. Effect of early metoprolol on infarct size in ST-segment-elevation myocardial infarction patients undergoing primary percutaneous coronary intervention: the Effect of Metoprolol in Cardioprotection During an Acute Myocardial Infarction (METOCARD-CNIC) trial. Circulation. 2013;128(14):1495-503. doi:10.1161/CIRCULATIONAHA.113.003653.

37. Pizarro G, Fernandez-Friera L, Fuster V, Fernandez-Jimenez R, Garcia-Ruiz JM, Garcia-Alvarez A et al. Long-term benefit of early pre-reperfusion metoprolol administration in patients with acute myocardial infarction: results from the METOCARD-CNIC trial (Effect of Metoprolol in Cardioprotection During an Acute Myocardial Infarction). Journal of the American College of Cardiology. 2014;63(22):2356-62. doi:10.1016/j.jacc.2014.03.014.

38. Mewton N, Thibault H, Roubille F, Lairez O, Rioufol G, Sportouch C et al. Postconditioning attenuates no-reflow in STEMI patients. Basic research in cardiology. 2013;108(6):383. doi:10.1007/s00395-013-0383-8.

39. Woo JS, Kim W, Ha SJ, Kim JB, Kim SJ, Kim WS et al. Cardioprotective effects of exenatide in patients with ST-segment-elevation myocardial infarction undergoing primary percutaneous coronary intervention: results of exenatide myocardial protection in revascularization study. Arteriosclerosis, thrombosis, and vascular biology. 2013;33(9):2252-60. doi:10.1161/ATVBAHA.113.301586.

40. Yoon CH, Chung WY, Suh JW, Cho YS, Youn TJ, Chun EJ et al. Distal protection device aggravated microvascular obstruction evaluated by cardiac MR after primary percutaneous intervention for ST-elevation myocardial infarction. International journal of cardiology. 2013;167(5):2002-7. doi:10.1016/j.ijcard.2012.05.029.

41. Garcia-Dorado D, Garcia-Del-Blanco B, Otaegui I, Rodriguez-Palomares J, Pineda V, Gimeno F et al. Intracoronary injection of adenosine before reperfusion in patients with ST-segment elevation myocardial infarction: A randomized controlled clinical trial. International journal of cardiology. 2014;177(3):935-41. doi:10.1016/j.ijcard.2014.09.203.

42. Jones DA, Pellaton C, Velmurugan S, Rathod KS, Andiapen M, Antoniou S et al. Randomized phase 2 trial of intracoronary nitrite during acute myocardial infarction. Circulation research. 2015;116(3):437-47. doi:10.1161/CIRCRESAHA.116.305082.

43. Ko YG, Won H, Shin DH, Kim JS, Kim BK, Choi D et al. Efficacy of early intensive rosuvastatin therapy in patients with ST-segment elevation myocardial infarction undergoing primary percutaneous coronary intervention (ROSEMARY Study). The American journal of cardiology. 2014;114(1):29-35. doi:10.1016/j.amjcard.2014.03.059.

44. Limalanathan S, Andersen GO, Klow NE, Abdelnoor M, Hoffmann P, Eritsland J. Effect of ischemic postconditioning on infarct size in patients with ST-elevation myocardial infarction treated by primary PCI results of the POSTEMI (POstconditioning in ST-Elevation Myocardial Infarction) randomized trial. Journal of the American Heart Association. 2014;3(2):e000679. doi:10.1161/JAHA.113.000679.

45. Siddiqi N, Neil C, Bruce M, MacLennan G, Cotton S, Papadopoulou S et al. Intravenous sodium nitrite in acute ST-elevation myocardial infarction: a randomized controlled trial (NIAMI). European heart journal. 2014;35(19):1255-62. doi:10.1093/eurheartj/ehu096.

46. Waltenberger J, Gelissen M, Bekkers SC, Vainer J, van Ommen V, Eerens F et al. Clinical pacing post-conditioning during revascularization after AMI. JACC Cardiovascular imaging. 2014;7(6):620-6. doi:10.1016/j.jcmg.2014.01.017.

47. Carrick D, Oldroyd KG, McEntegart M, Haig C, Petrie MC, Eteiba H et al. A randomized trial of deferred stenting versus immediate stenting to prevent no- or slow-reflow in acute ST-segment elevation myocardial infarction (DEFER-STEMI). Journal of the American College of Cardiology. 2014;63(20):2088-98. doi:10.1016/j.jacc.2014.02.530.

48. Roubille F, Mewton N, Elbaz M, Roth O, Prunier F, Cung TT et al. No post-conditioning in the human heart with thrombolysis in myocardial infarction flow 2-3 on admission. European heart journal. 2014;35(25):1675-82. doi:10.1093/eurheartj/ehu054.

49. Erlinge D, Gotberg M, Lang I, Holzer M, Noc M, Clemmensen P et al. Rapid endovascular catheter core cooling combined with cold saline as an adjunct to percutaneous coronary intervention for the treatment of acute myocardial infarction. The CHILL-MI trial: a randomized controlled study of the use of central venous catheter core cooling combined with cold saline as an adjunct to percutaneous coronary intervention for the treatment of acute myocardial infarction. Journal of the American College of Cardiology. 2014;63(18):1857-65. doi:10.1016/j.jacc.2013.12.027.

50. Atar D, Arheden H, Berdeaux A, Bonnet JL, Carlsson M, Clemmensen P et al. Effect of intravenous TRO40303 as an adjunct to primary percutaneous coronary intervention for acute ST-elevation myocardial infarction: MITOCARE study results. European heart journal. 2015;36(2):112-9. doi:10.1093/eurheartj/ehu331.

51. White SK, Frohlich GM, Sado DM, Maestrini V, Fontana M, Treibel TA et al. Remote ischemic conditioning reduces myocardial infarct size and edema in patients with ST-segment elevation myocardial infarction. JACC Cardiovascular interventions. 2015;8(1 Pt B):178-88. doi:10.1016/j.jcin.2014.05.015.

52. Kim EK, Hahn JY, Song YB, Lee SC, Choi JH, Choi SH et al. Effect of ischemic postconditioning on myocardial salvage in patients undergoing primary percutaneous coronary intervention for ST-segment elevation myocardial infarction: cardiac magnetic resonance substudy of the POST randomized trial. The international journal of cardiovascular imaging. 2015;31(3):629-37. doi:10.1007/s10554-015-0589-y.

53. Nichol G, Strickland W, Shavelle D, Maehara A, Ben-Yehuda O, Genereux P et al. Prospective, multicenter, randomized, controlled pilot trial of peritoneal hypothermia in patients with ST-segment- elevation myocardial infarction. Circulation Cardiovascular interventions. 2015;8(3):e001965. doi:10.1161/CIRCINTERVENTIONS.114.001965.

54. Stub D, Smith K, Bernard S, Nehme Z, Stephenson M, Bray JE et al. Air Versus Oxygen in ST-Segment Elevation Myocardial Infarction. Circulation. 2015. doi:10.1161/CIRCULATIONAHA.114.014494.

55. Hoole SP, Jaworski C, Brown AJ, McCormick LM, Agrawal B, Clarke SC et al. Serial assessment of the index of microcirculatory resistance during primary percutaneous coronary intervention comparing manual aspiration catheter thrombectomy with balloon angioplasty (IMPACT study): a randomised controlled pilot study. Open Heart. 2015;2(1):e000238. doi:10.1136/openhrt-2015-000238.

56. Kim EK, Hahn JY, Song YB, Chang SA, Choi JH, Choi SH et al. Effects of high-dose atorvastatin pretreatment in patients with ST-segment elevation myocardial infarction undergoing primary percutaneous coronary intervention: a cardiac magnetic resonance study. Journal of Korean medical science. 2015;30(4):435-41. doi:10.3346/jkms.2015.30.4.435.

57. McCann GP, Khan JN, Greenwood JP, Nazir S, Dalby M, Curzen N et al. Complete Versus Lesion-Only Primary PCI: The Randomized Cardiovascular MR CvLPRIT Substudy. Journal of the American College of Cardiology. 2015;66(24):2713-24. doi:10.1016/j.jacc.2015.09.099.

58. Deftereos S, Giannopoulos G, Angelidis C, Alexopoulos N, Filippatos G, Papoutsidakis N et al. Anti-Inflammatory Treatment With Colchicine in Acute Myocardial Infarction: A Pilot Study. Circulation. 2015;132(15):1395-403. doi:10.1161/CIRCULATIONAHA.115.017611.

59. Eitel I, Stiermaier T, Rommel KP, Fuernau G, Sandri M, Mangner N et al. Cardioprotection by combined intrahospital remote ischaemic perconditioning and postconditioning in ST-elevation myocardial infarction: the randomized LIPSIA CONDITIONING trial. European heart journal. 2015;36(44):3049-57. doi:10.1093/eurheartj/ehv463.

60. Nazir SA, McCann GP, Greenwood JP, Kunadian V, Khan JN, Mahmoud IZ et al. Strategies to attenuate micro-vascular obstruction during P-PCI: the randomized reperfusion facilitated by local adjunctive therapy in ST-elevation myocardial infarction trial. European heart journal. 2016;37(24):1910-9. doi:10.1093/eurheartj/ehw136.

61. Roolvink V, Ibanez B, Ottervanger JP, Pizarro G, van Royen N, Mateos A et al. Early Intravenous Beta-Blockers in Patients With ST-Segment Elevation Myocardial Infarction Before Primary Percutaneous Coronary Intervention. Journal of the American College of Cardiology. 2016;67(23):2705-15. doi:10.1016/j.jacc.2016.03.522.

62. Roos ST, Timmers L, Biesbroek PS, Nijveldt R, Kamp O, van Rossum AC et al. No benefit of additional treatment with exenatide in patients with an acute myocardial infarction. International journal of cardiology. 2016;220:809-14. doi:10.1016/j.ijcard.2016.06.283.

63. Belle L, Motreff P, Mangin L, Range G, Marcaggi X, Marie A et al. Comparison of Immediate With Delayed Stenting Using the Minimalist Immediate Mechanical Intervention Approach in Acute ST-Segment-Elevation Myocardial Infarction: The MIMI Study. Circulation Cardiovascular interventions. 2016;9(3):e003388. doi:10.1161/CIRCINTERVENTIONS.115.003388.

64. Liu Z, Zhao L, Hong D, Gao J. Remote ischaemic preconditioning reduces myocardial ischaemic reperfusion injury in patients with ST-elevation myocardial infarction undergoing primary percutaneous coronary intervention. Acta Cardiol. 2016;71(5):596-603. doi:10.2143/AC.71.5.3167504.

65. Dominguez-Rodriguez A, Abreu-Gonzalez P, de la Torre-Hernandez JM, Gonzalez-Gonzalez J, Garcia-Camarero T, Consuegra-Sanchez L et al. Effect of intravenous and intracoronary melatonin as an adjunct to primary percutaneous coronary intervention for acute ST-elevation myocardial infarction: results of the Melatonin Adjunct in the acute myocaRdial Infarction treated with Angioplasty (MARIA) trial. J Pineal Res. 2016. doi:10.1111/jpi.12374.

66. Verouhis D, Sorensson P, Gourine A, Henareh L, Persson J, Saleh N et al. Effect of remote ischemic conditioning on infarct size in patients with anterior ST-elevation myocardial infarction. Am Heart J. 2016;181:66-73. doi:10.1016/j.ahj.2016.08.004.
